# Community-based Health Data Cooperatives Towards Improving the Immigrant Community Health: A Scoping Review to Inform Policy and Practice

**DOI:** 10.23889/ijpds.v5i1.1158

**Published:** 2020-06-23

**Authors:** I Naeem, AKMN Nurul, M Vaska, S Goopy, R Rashid, A Kassan, F Aghajafari, I Ferrer, A Kazi, I Sadi, M O'Beirne, C Leduc, TC Turin

**Affiliations:** 1 Department of Community Health Sciences, University of Calgary, Calgary, Alberta, Canada; 2 Department of Family Medicine, University of Calgary, Calgary, Alberta, Canada; 3 Department of Economics, University of Calgary, Calgary, Alberta, Canada; 4 Tom Baker Cancer Centre, Foothills Medical Centre, University of Calgary, Calgary, Alberta, Canada; 5 Faculty of Nursing, University of Calgary, Calgary, Alberta, Canada; 6 Werklund School of Education, University of Calgary, Calgary, Alberta, Canada; 7 Faculty of Social Work, University of Calgary, Calgary, Alberta, Canada; 8 Citizen Researcher, Calgary, Alberta, Canada

## Abstract

**Background:**

In the case of immigrant health and wellness, data are the key limiting factor, where comprehensive national knowledge on immigrant health and health service utilisation is limited. New data and data silos are an inherent response to the increase in technology in the collection and storage of data. The Health Data Cooperative (HDC) model allows members to contribute, store, and manage their health-related information, and members are the rightful data owners and decision-makers to data sharing (e g. research communities, commercial entities, government bodies).

**Objective:**

This review attempts to scope the literature on HDC and fulfill the following objectives: 1) identify and describe the type of literature that is available on the HDC model; 2) describe the key themes related to HDCs; and 3) describe the benefits and challenges related to the HDC model.

**Methods:**

We conducted a scoping review using the five-stage framework outlined by Arskey and O’Malley to systematically map literature on HDCs using two search streams: 1) a database and grey literature search; and 2) an internet search. We included all English records that discussed health data cooperative and related key terms. We used a thematic analysis to collate information into comprehensive themes.

**Results:**

Through a comprehensive screening process, we found 22 database and grey literature records, and 13 Internet search records. Three major themes that are important to stakeholders include data ownership, data security, and data flow and infrastructure.

**Conclusions:**

The results of this study are an informative first step to the study of the HDC model, or an establishment of a HDC in immigrant communities.

**Key words:**

community health, health data, cooperative, and citizen data empowermen

## Introduction

Canada is becoming an increasingly multicultural society, welcoming 250,000 immigrants each year, and composing 20% of the Canadian population [1]. Immigrant communities add to the economic, labor, and cultural diversity of Canada. An immigrant, as defined by Statistics Canada, is any person residing in Canada who was born outside of the country, excluding temporary foreign workers, Canadian citizens born outside Canada, and those with student working visas [2]. Comprehensive national knowledge on immigrant health and health service utilisation is limited to allow for meaningful comparisons either within immigrant sub-groups or between immigrants and the native-born population [3]. Key knowledge gaps in immigrant health include long-term health outcomes, preventable conditions, and chronic disease outcomes, especially amongst subgroups such as refugee and non-European immigrants [4]. Immigrants comprise relatively small portions of the population given that approximately 18% of the Canadian population were born outside of the country. However, meaningful comparisons and generalisable results require large-scale studies that sample an effective number of immigrants, which is often not done with studies using statistics Canada data sets of national population health surveys. This includes the National Population Health Survey (NPHS), Canadian Community Health Survey (CCHS), the National Longitudinal Survey of Children and Youth (NLSCY), the General Social Survey (GSS) [5,6].

In the case of immigrant health, data is the key limiting factor, where comprehensive national knowledge on immigrant health and health service utilisation is limited. There has been a push to increase available immigrant data through linking Canadian immigrant databases to administrative databases in order to explore patterns of mortality, cancer incidence, hospitalizations, physician claims, prescription medication use, and socio-demographic factors of health [4]. However, there is a lack of immigrant data to carry out meaningful comparisons either within immigrant sub-groups or between immigrants and the native-born population. Indeed, to understand how the process of immigration and acculturation affect health, data specifically on immigrant are needed. Further, immigrant groups have culturally based understandings of health and illness, or differences in definition of appropriate ways to treat illness. Health data that is sensitive to these differences would allow for a deeper understanding of health in this group, and may prove fruitful for public health research, policy, and tailored treatments [7].

### 'Health Data Cooperatives': Are they the solution?

The Health data co-operative (HDC) model is that of a health data bank that is established as an approach to make health data available to society. The cooperative members can contribute to, store, and manage their health-related information and are the rightful data owners and decision-makers to data sharing (e g. research communities, commercial entities, government bodies). Individuals and the community at large may profit from the value of the data both financially and/or through in-kind contributions by providing access to the data to health-related establishments, including research organisations and government services. Combining the HDC members’ demographic, socioeconomic and lifestyle information through a community owned HDC is expected to create tremendous potential for community mobilisation and empowerment [8].

Further, new data and data silos are an inherent response to the increase in technology. For example, gene sequencing gives us access to personal information that may inform the effectiveness of drugs, and health risk factors to certain disease. Further, with the rise of smartphones, there are more than 40,000 health applications that allow for longitudinal monitoring of health without a visit to the doctor’s office. These examples, along with physician offices, hospitals, and laboratories create data silos, where the data becomes isolated in a physically, judicially, or intellectual defined area, and is unavailable for integration across multiple platforms. This result is a lack of autonomy and empowerment in the control of data by citizens, and unnecessary costs by the healthcare systems due to the inaccessibility of this data for analysis and research. This can be especially useful for under-represented population such as immigrants [9].

A health data cooperative is a collective where health related data are integrated, stored, used, and shared under the control of the cooperative members. The brackets in the following examples simply show the type of data a potential cooperative can host, along with potential stakeholders who can use the data. Most HDCs follow a similar structure that starts with 1) members who are any persons who wish to join a cooperative and contribute their data (e.g. health condition, lab results, hospital admission information, 23andMe, data generated from apps, doctor offices, clinic visits); 2) data storage safe space; 3) data sharing (e.g. authorities, doctors, research, patient group) [10]. The HDC model has been common in Europe, with the establishment of the Federation of National HDCs. This federation shares a common IT structure and common data storage. Further, the federation benefits from political support from Swiss stakeholders and truly encapsulates all that HDCs stand for, including being citizen-owned and citizen-centered and secure in storage and management of data [10].

### Research Objectives

The need for immigrant groups to contribute to an HDC model is an important target for public health research. The integration of and access to immigrant specific health data can empower individuals to better manage their health, help contribute to the care of family and friends, help professionals commission the most effective and efficient interventions to benefit the community, allow researchers to develop next generation medicine, and enable the innovation of health technology [10].

Given the importance of data in immigrant health research, it is imperative to scope the literature to understand the complexities of HDC at the national and international level. To systematically map the available literature on the topic of HDCs, the current scoping review will aim to: 1) identify and describe the type of literature that is available on the HDC model; 2) describe the key themes related to HDCs; and 3) describe the benefits and challenges of the HDC model. This scoping review will serve to inform the next stages of research and community engagement to develop a community-based health data cooperative in immigrant communities in Canada. To our knowledge, this is the first review undertaken to synthesize HDC literature.

**Table 1: Keyword MeSH terms used for search in databases:** 

Keywords for health data:

“Personal Health Record*”[Keyword]; PHR [Keyword]; Data [Keyword]; “data set* [Keyword]; databank [Keyword]; “data bank” [Keyword]; Health [Keyword, MeSH]; Crowd-sourced [Keyword]; crowdsourcing [Keyword, MeSH]; “health trust” [Keyword]; “health data [Keyword]; “Electronic Health Record*” [Keyword]; EHR [Keyword]; “Health Information Exchange *” [Keyword]
Keywords for cooperative:

“data cooperative” [Keyword]; Cooperative* [Keyword]; “cooperative business model*” [Keyword]; “cooperative model*” [Keyword]; Citizen-generated [Keyword]; consumer participation [MeSH]; client-generated [Keyword]; people-generated [Keyword]; public-generated [Keyword]; Citizen-driven [Keyword]; client-driven [Keyword]; people-drive [Keyword]; public-driven [Keyword]; citizen-owned [Keyword]; citizen-controlled [Keyword]; “citizen science” [Keyword]; citizen-centric [Keyword]; client-centric [Keyword]; person-centric [Keyword]; people-centric [Keyword]; “citizen empowerment” [Keyword]; “client empowerment” [Keyword]; “people empowerment” [Keyword]; “public empowerment” [Keyword]; Citizen* [Keyword]; client* [Keyword]; public [Keyword]; person* [Keyword] persons [MeSH]; Empowerment [Keyword]; power (psychology) [MeSH]; self-empowerment [Keyword]; Ownership [Keyword, MeSH]

## Methods

We conducted a scoping review to systematically map and synthesize the information available on HDCs utilising the reporting guidelines of the PRISMA extension for scoping reviews (PRISMA- ScR) [11] (Supplementary Appendix 1). Further, we followed the framework outlined by Arksey and O’Malley in their methodological paper on scoping reviews [12]. In their paper, the authors discuss the purpose of scoping reviews as an attempt to examine the extent, range, and nature of the research question, without describing the findings of the studies in detail or doing an assessment of quality. The same approach is taken with this review, using the Arksey and O’Malley five-stage framework.

### Stage 1. Identifying the research question

An effective scoping review requires a research question situated within a specific area, but it must also remain broad so as not to exclude potentially useful literature. For this review, we posed the following non-limiting questions: 1) what are the major themes associated with HDCs; and 2) what are the benefits and challenges related to the HDC model.

### Stage 2. Identifying relevant studies

Academic published literature searches were conducted using bibliographic databases presented in Table 1 to identify relevant records. An experienced librarian (MV), oversaw the development and execution of the database search strategies, which included a predefined list of keywords and medical subject heading (MeSH) terms (see Table 1) (see Supplementary Appendix 2 for MEDLINE (Ovid) search strategy). For grey literature, our search strategy included electronic institutional repositories, Canadian and international professional and government websites, online literature sites, and a manual review of reference lists of relevant publications (see Table 2).

**Table 2: Database search list:** 

Health sciences: MEDLINE (Ovid) EMBASE PsycINFO EBM Reviews HealthSTAR PubMed PubMed Central CINAHL MEDLINE (Ebsco) Social sciences: Psychology & Behavioral Sciences Collection Social Science Data Archive SocIndex with FullText Sociological Abstracts Social Work Abstracts Business: Canadian Public Policy Collection Business Source Complete Multi-disciplinary: Web of Science Education Research Complete ERIC Urban Studies Abstracts Scopus Canadian Research Index LegalTrac Political science: International Political Science Abstracts PAIS Index Academic-focused search engines: Google Scholar	Repositories/theses: ProQuest (theses and dissertations) OAISter (WorldCat) Health sciences: Health Sciences Online (HSO) Turning Research into Practice (TRIP) Canadian Institutes of Health Research (CIHR) Canadian Institute for Health Information (CIHI) Public Health Agency of Canada (PHAC) Health Canada National Institutes of Health (NIH) World Health Organization (WHO) National Health Services (NHS) Alberta Health Services (AHS) Social sciences: International Federation of Social Science Organizations (IFSSO) Federation of Data Organizations for Social Science (IFDO) Consortium of Social Science Associations (COSSA) Organization for Social Science Research in Eastern and Southern Africa (OSSREA) International Organization of Social Sciences and Behavioral Research (IOSSBR) Other: Data Assurance & Analytics OpenDataBC Data Protection Law and the Ethical Use of Analytics Canadian Public Legal Education (CPLE) organizations Accessing Health and Health-Related Data in Canada DAMA International: Global Data Management Community National Association of Health Data Organizations (NAHDO) HEIMDALL: Multi-Hazard Cooperative Management Tool for Data Exchange, Response Planning, and Scenario Building

We also conducted a literature search using the three most popular search engines, Google [13], Yahoo! [14], and Bing [15] as these search engines represent more than 96.4% of all search engines worldwide. In addition, meta search engines that blend Web results from Google, Yahoo and Bing, namely MetaCrawler [16] and Monster Crawler [17] were consulted as well. The Internet was used both to identify relevant Web-based information on HDCs and to identify references to non-Web-based information. Our Web search included all relevant webpages from government and non-government organisations, news sites, blogs, discussion boards, social media platforms etc. We executed searches using the following keyword strings: (1) health data cooperative, (2) patient data cooperative and (3) personal health records. Adhering to the recommended search methodology of the Canadian Institute for Health Information (CIHI, 2011), only the first ten pages of the search were utilised for screening. Search results were downloaded and managed in EndNote software (Clarivate Analytics, Philadelphia, Pennsylvania, USA).

### Stage 3. Study Selection

We limited studies to those published in the English language, with no publication date limits. For the grey literature and database search, after removal of duplicates, IN and HN reviewed titles and abstracts for each paper or document for inclusion. Abstracts were classified as relevant, potentially relevant or not relevant to HDCs. Relevant records were those that directly discussed the HDC model or health data, data sharing, stakeholder engagement in data, and data ownership. Records were excluded if they did not discuss any of the concepts above. Abstracts that do not provide enough information on outcomes to determine eligibility were included for further review. Full texts were obtained of the abstracts that met eligibility criteria and were read, reviewed and re-examined for relevance. Two researchers reviewed the full text of the remaining papers to determine eligibility using the same criteria listed above. If no agreement was reached between the two researchers, TCT arbitrated.

Owing to the dynamic nature of the Internet, the screening and full review of webpages identified in the internet search was conducted concurrently. NH performed the first search string, archiving the list of the first ten pages (i.e. Web address, page title, brief description and date searched) and classified each as potentially useful or not useful based on the same criteria listed above for the database and grey literature search. The reviewer then opened all pages considered potentially useful, archived each full page and, should the page contain information about an HDC, classified that tool as eligible or ineligible for inclusion. Reasons for exclusion were recorded. The second reviewer (IN) then independently opened the archives created by the primary reviewer and assess random selections of ten percent of the websites classified by the primary reviewer as eligible and ten percent of those classified as ineligible. The second reviewer also archived the full page of each website opened for assessment (i.e., included and excluded) in case the content had been modified since the primary reviewer undertook screening and full review. If no agreement was reached between the two researchers, TCT arbitrated.

### Stage 4: Charting the Data

Information was extracted on the citation, study location, study objective, the health-data-related variable, how the cooperative was established, the main outcome variables, how the outcome variables were measured, and benefits and challenges of HDC. Further, due to the scoping nature of this review, additional descriptive information was collected for selected records including country of origin, author affiliation, and target audience. Two reviewers (IN and NH) independently carried out the data extraction, and discrepancies were resolved by consensus.

### Stage 5: Collating, Summarising and reporting the Results

To explore our first research question, we conducted a content analyses of the abstracted data from the records to discover, and generated themes that were adjusted iteratively. For our second research question, the abstracted information on benefits and challenges related to HDC models was stratified by these themes (Tables 3 & 4).

**Table 3: Descriptive data and major themes of included studies.** 

Author	Year	Location	Publication Type	Author affiliation, Target audience	Major themes related to HDC
DATABASE SEARCH					

Contreras et al.	2018	UK	Letter to editor	Academic, Academia	Who owns the data by law? Will the HDC replace or supplement existing ideas? Third-party, patient-controlled compilations of health data, such as mobile health records already exist – how will they be incorporated into HDC?
Grumbach et al.	2014	USA	Research Article	Academic, Academia	Acquiring an electronic health for a healthcare cooperative will need to create functional patient registries A primary care cooperative extension service would provide technical assistance in the implementation of chronic care models, advanced access scheduling, group medical visits, and similar innovations.
Hafen et al.	2014	Switzerland	Research Article	Academic, Academia/policy	Precision medicine and personalized health are related. already 40000 health related apps that collect patient data and can contribute towards precision medicine. Personal data is a new asset, patients have limited access due to i) decentralized storage, ii) data protection laws
Mahlmann et al.	2018	Switzerland	Research Article	Academic, Academia /policy/Public health workers	New data sources are emerging, such as: lifestyle data, quantified self movement, demographic data etc. The private sector is rapidly adopting data strategies No integration of big data into public health policies Data are not interlinked - inability to realize the potentials of data from apps etc.
Mikk et al.	2017	USA	Commentary	Academic, Academia	Commentary is in response to a comment that individuals who have control over their longitudinal data are less likely to share it. Authors prove that individuals are more likely to share their health data for research as compared to their physicians.
Tracy et al.	2004	Canada	Research Article	Academic, Academia	Most participants possessed extremely limited knowledge of how their public health information is collected, used, and disclosed; They don’t have control over collection, use and disclosure of PHR; scared of privacy; new tool can increase security; have mistrust about the protection about their privacy; HCID would prevent breaches of privacy; Access to medical records is generally considered appropriate after consent has been obtained but there is lack of clarity as to whether express consent is required for each and every use; Individuals want privacy of own data (solved by governance) but public benefit from data (tools needed)
Van Roessel et al.	2018	Switzerland	Research Article	Academic, Academia/policy	HDC is citizen owned, equal property of members; not for profit; revenues will be reinvested; Because of technological advances, the amount of available personal data will expand considerably; The willingness to share personal health information increases when individuals have control over their own data and the information is anonymous
Vayena et al.	2017	Switzerland	Review	Academic, Academia/Policy /Public health workers	Traditional means of control: Data control is conducive to transparency, accountability, and trust which includes informed consent; professional confidentiality; anonymization Emerging models of Control include control over data access, control over data uses, and governance
Montgomery J.	2017	USA	Review	Academic, Academia/Policy /Public health workers	Health information as the private property of patients It is hard to justify on the traditional ‘labour theory’ of ownership. That approach would instead suggest that health information derived from patients should be owned by health professionals (or more plausibly the health systems for whom they work). However, giving either patients or individual professionals the right to extract ransom payments from those seeking to use genomic science to provide personalised medicine enables them to appropriate to themselves material that is biologically common to others.
Blasimme et al.	2018	Netherland	Commentary	Academic, Academia/Policy /Public Health workers	Informed consent; Privacy issues; governance, and readiness
Dorey et al.	2018	Netherland	Research Article	Academic, Academia, Policy, Public Health workers	Qualitative study gain an in-depth understanding of the awareness of possible ethical risks and corresponding obligations among those who are involved in projects using patient data, i.e. healthcare professionals, regulators and policy makers. Interviewees pointed out the risks of collecting the wrong data, or in the wrong way and generating waste; Respondent attitude: a) all interviewees recognized patient rights to know, to protect privacy and to own their data. However, their attitude regarding patient information indicated some discrepancies with this position; b) they recognized that, depending on their purpose, all health stakeholders could benefit from CRGs; d) it was suggested that physicians needed to be better trained in information technologies and public health sciences; e) Most interviewees supported public governance to serve public interest
King et al.	2016	USA	Research Article	Non-academic, policy	Community health record should focus: 1) enable meaningful collaboration, 2) facilitate a shared approach, 3) build workforce and infrastructure capacity, and 4) establish a new way of doing business that enables the transformation of community health data into information and information into knowledge to aid decision makers in collectively improving population health.
Torres et al.	2014	USA	Research Article	Academic, Policy	Health care market characteristics and their impacts on data sharing within and across Communities; Provided strategies that selected communities employed to build and strengthen their data sharing infrastructure. Also provided information on usability and integration of electronic data exchange into workflows
Allen et al.	2014	USA	Case Study	Non-academic, Policy	Can drive improvements in health and health care by increasing the accuracy, accessibility, and utility of patient information

GREY LITERATURE SEARCH					

Denise et al.	2012	USA	Policy report	Academic, Policy	This report discusses a common community data set Today, discharge data provides the full community of users with information that is relatively current, has provider identifiers, and is cost efficient. The information is used in public displays, such as websites, dynamic web query systems, and in traditional reports. It provides a broad array of information not found in individual registries and is more cost efficient to collect than other sources. It can also be de-identified to allow broader use, than is possible with other clinical data sources. Because they are widely available and broadly used, hospital discharge data could serve as the backbone for a hybrid EMR/discharge “package” of information. Statewide discharge data combined with clinical data in an EMR can supply both the numerator and denominator for examining outcomes of care and cost effectiveness of treatments. In addition, the common structure and relative uniformity of hospital discharge data across providers and states, allows for regional and national comparisons.
Nadeau, E.G.	2010	USA	Report	Academic, Academia	The key features of national Cooperative business Association in community health mobilization model in western Kenya are summarized. Autonomous and democratically run local organizations. Additional community-based organizations. Local residents form women’s groups, youth groups, HIV-AIDS support groups and other organizations that carry out their own health education, health services, and economic development activities.
Future Care Capital	2017	UK	Company Report	Non-academic, Policy	A growing number of organisations are making progress in integrating health and care record data at the local level, but the complexities surrounding Information Governance (IG) modelling are impacting associated timescales as well as the potential for such data to be put to beneficial secondary uses. The process took those this report interviewed up to twelve months to finalise, and none plans to integrate substantial information from social care home providers at present, which would almost certainly take more time. Only one of the interviewees used the data collected for purposes other than direct care and provided third party access for research based upon informed consent.
Ken, T.	2015	USA	News article	Academic, Academia	Article describes the struggle in the ownership of patient medical data Themes are presented about data ownership and how medical practices can be adjusted to meet ownership laws.

Website Name, URL	Location	Purpose of Website	Private or Academic	Themes
INTERNET SEARCH					

A healthcare Startup to provide patient perspective adopts co-op model, https://medcitynews.com/2018/04/healthcare-startup-provide-patient-perspective-adopts-co-op-model/	Europe	News	Private	From the perspective of Savvy Cooperative CEO and founder Jen Horonjeff, if pharma companies, healthcare organizations, and technology companies want a patient’s perspective to create better treatments and solutions, then they should pay patients, not just the company providing access. Savvy Cooperative is set up as a patient co-operative.
Are digital data co-ops an alternative to the commercialisation of health? https://thisisnotasociology.blog/2017/02/27/are-digital-data-co-ops-an-alternative-to-the-commercialisation-of-health	USA	Blog	Private	The Coop will mostly generate funding through fees paid for access to the data by researchers or companies with profits reinvested in the organisation (there will not be shareholders). Individuals will be able to refuse access to their data to particular organizations or companies and the membership will be able to vote on who is accepted as a client.
Data Cooperatives - P2P Foundation, http://wiki.p2pfoundation.net/Data_Cooperatives	Europe	Research	Academic	You manage the key to your data. The cooperative will take care of the collective utilization of that data for you in the interest of you. The proceeds will be reinvested in a new health economy: an economy that has an interest in your health instead of your illness
Holland Health Data Cooperative | LinkedIn, https://www.linkedin.com/company/holland-health-data-cooperativ	Europe	Corporate	Private	The diversity of personal health record Platforms (PHR) includes: Business driven initiatives from the IT giants, sensor driven initiatives. Access through web and mobile apps (mHealth), data visualization (timelines, graphs, calendar views, ...), data-sharing with friends, health professionals, coaches
Swiss Data alliance, https://swiss-data-alliance.squarespace.com	Europe	Presentation		The diversity of personal health record Platforms (PHR) includes: Business driven initiatives from the IT giants, sensor driven initiatives. Access through web and mobile apps (mHealth), data visualization (timelines, graphs, calendar views, ...), data-sharing with friends, health professionals, coaches
MIDATA.COOPs – Personal (Health) Data Cooperatives, https://www.midata.coop/	Europe	Corporate	Private	MIDATA outlines the Primary and Secondary Use of Data Primary use # E.g. Patient – physician interaction # E.g. Citizen – commercial provider (according to digital agreements with App providers and retail companies (e.g. Micros Cumulus) Secondary use # Value generated through aggregation (e.g. advertising, clinical research, public health) # $ 1 Bn personal data economy (health data not included) builds on secondary use # Currently citizens have not control and little benefit from secondary use
Patient Ownership creating the business Environment http://www.ourhdc.com/files/77395669.pdf	USA	Corporate	Private	The current siloed structure of US health data repositories requires researchers to access a variety of proprietary databases, controlled by individual healthcare systems providers or to request access to health providers' patients for randomized double-blind samples.
The future of your health data, http://maneeshjuneja.com/blog/2014/2/12/the-future-of-your-health-data	USA	Blog	Private	Our Health Data Co-Operative is in the US and has recently been recognised by the White House as playing a role in promoting "Data to Knowledge to Action". The founder, Patrick Grant, states, "Our Health Data Cooperative is built on the premise that Patients should benefit economically from access by third parties to their health information." HealthBank: A patient data co-operative based in Switzerland but aiming to build a global secure depository for patient data. Their website talks of patients having "a HealthBank account, to store, access, manage and share their health data. And users can earn financial and other returns on their health data, similar to receiving returns from a bank account."
The Personal Data Economy – A Cooperative Approach - Inspire2Live, http://inspire2live.org/wp-content/uploads/10.-Ernst-Hafen-Personal-Data-Economy-a-cooperative-approach.pdf	Europe	Research	Academic	The Challenge: Rebalancing the socioeconomic asymmetry in a data-driven economy.
This co-op lets patients monetize their own health data - Fast Company, https://www.fastcompany.com/90207550/this-co-op-lets-patients-monetize-their-own-health-data	USA	News	Private	In 2016, Horonjeff, along with her co-founder Ronnie Sharpe, who grew up with cystic fibrosis and founded a social network for others with the diseases, started Savvy, a platform to bridge the gap between patients and practitioners. The platform officially launched in the fall of 2017, and recently became a public benefit corporation.
Towards a European Ecosystem for Healthcare Data – Digital, https://digitalenlightenment.org/system/files/def_healthcare_data_management_report_oct2017_v3.5.pdf	Europe	Research	Academic	The Data Commons Co-op greases the flow of data between communities in the cooperative, solidarity, new, call-it-what-you-will economy. The co-op not only serves these communities, it is owned by them.
Data Commons Cooperative, https://datacommons.coop/	Australia	Corporate	Private	The Data Commons Co-op greases the flow of data between communities in the cooperative, solidarity, new, call-it-what-you-will economy. The co-op not only serves these communities, it is owned by them.
Digital Health CRC – Home, https://www.digitalhealthcrc.com/	USA	Corporate	Private	This corporation is developing a unique, multidisciplinary, collaborative taskforce of research, clinical, industry, government and educational organizations to focus research and development on combining individual and collective expertise with data, information and telecommunication technologies. Company themes: Empowering consumers, improving understanding of health risks in individuals and communities, supporting clinical practice, improving system efficiency by joining up data, improving access to quality care The desired outcomes Our collaborative approach will: improve the health and wellbeing of hundreds of thousands of Australians, improve value of care, create a new digital workforce through at least 1000 new jobs in digital health and related industries.

**Table 4: Reported benefits and challenges of the HDC model:** 

Author	Benefits	Challenges
DATABASE SEARCH					

Contreras et al. 2018	HDC creates a longitudinal health data from various care settings HDC would supplement records currently available HDC is controlled by patients through contract law and other mechanisms	Individual ownership of data is contrary to well-established legal precedent in nations like US, UK Patient might not want to share certain data
Grumbach et al. 2009	Healthcare cooperatives for chronic disease can involve multiple stakeholders, including primary care physicians, beneficiaries, community members health departments, and social services and universities. The healthcare cooperatives should be organized around a state of regional hub, which in turn support county agency offices.	Staff of a health care cooperative would need constant and standardized training. The cooperative services would need to be delivered by an agency within the US department of Health and Human Services, which may cause loss of autonomy on part of the communities.
Hafen et al. 2004	Data can be stored in "core" accessed by apps; used by researcher via big data analytics A federation can be created globally	No system can absolutely guarantee trust and transparency and data security. Data repositories should be certified by independent government regulatory bodies and good governance structures.
Mahlmann et al. 2017	With HDC, policy- making is data driven HDC allow the alignment of big data and advanced methods HDC could be applied as a tool for syndromic surveillance. HDC can lead to evidence-based prevention strategies Overcome the current practice of “one-size-fits-all” treatment, adjust therapies and prevention strategies to the individual’s needs	Complexity involved, and privacy and data protection issues. Data should remain in citizen ownership The framework should be based on trust, transparency, information, and openness. Finding a right balance between public health purposes, and personal privacy
Mikk et al. 2018	A longitudinal health data set for individuals can aggregate data from various care settings using common data elements. Data accessing can be updated or access in real time in a HDC model.	Scholars have reviewed the interplay of property law and privacy law on health records and health data, with the bottom line being that neither property nor privacy law is completely applicable to health
Tracy et al. 2004	Simple language should be used in apps or system; CSR or helpline is helpful;	Distrust, lack of respect, insufficient patient control of the process.
Van Roessel et al. 2017	HDC allows all types of data from a variety of sources to be included; Reduces cost of gathering data Third party needs to get consent, apps can be used; Cloud computing will enable accessing data anytime, connect patients and physician anytime; can be used for community education through chat, blogs etc	Reduced contact between patient and physician’s transparency about governance is key Physician cannot use the big data Absolute security is not guaranteed; Insufficient transparency may discourage patients, fear of anonymity breach may discourage; Not enough financial incentives; Third party can access data from other sources so commercial interest may be not much
Montgomery J. 2017		Treating health information as the private property of patients is hard to justify on the traditional ‘labour theory’ of ownership. Health information derived from patients should be owned by health professionals (or more plausibly the health systems for whom they work).
Blasimme et al. 2018	HDC can provide consent electronically Less empowered or historically underserved communities can organize to take control of their data and to voice their motivations, needs. Data cooperatives’ members can also allow their clinicians to access their data	Funding and the public’s commitment Healthy people may have less incentive Data access and portability rights Rights of data controller vs data owner
Dorey et al. 2018		Ethical awareness regarding the management of patient data in Swiss real-life settings where CRGs are decided, created, managed and used.
King et al. 2016	right to own data and privacy Potential for all stakeholder benefits Support for public governance of data to serve public interest	Written and informed consent is too burdensome for cooperative participants Risks associated with collecting the wrong data, or in the wrong way; generating waste
Torres et al. 2014	Can improve existing health care market dynamics, legal factors, and Communities’ respective visions for how the data would be used	Time and resources needed to lay the groundwork; Ability to customize IT systems and their capacity to help providers adapt Built in flexibility to help practices at all levels Ensure data sharing was not unnecessarily restricted while still protecting health information
Allen et al. 2014		Can be difficult to draft a data sharing agreement details of collection: use must be described, and an online data audit system must be developed to see who views data.

GREY LITERATURE SEARCH					

Love et al. 2012		Data collection and aggregation across providers is fraught with political and technical challenges. Provider resistance to initial aggregation of data Patient privacy and confidentiality concerns Data ownership and control issues arise when combining data across stewards
Nadeau, E.G. 2010	Implementation of the program as a clear step-by-step process for selecting local staff and volunteers, training them, organizing village and multi-village organizations Village led approach	Training of and groups mobilization for the program is difficult
Hartley et al. 2014	Establish a more formal collaborative to coordinate stakeholders cultivate public-private partnerships and promote precision medicine and personalized health	Establishing access and privacy standards for the protection and safety of patient data
Hafen E. 2014	Citizen-owned, citizen-centered Secure storage, management and sharing; citizens decide what data to share with whom (doctors, friends, research) Citizen decide how much information to receive (right not to know)	Data incompatibility Can have corporate feudalism developing
International Health Cooperative Organization, 2018	Participating stakeholders share a general-interest goal; common endeavour strengthens the links that cooperatives have with the local community and their ability to approximate its common good.	A progressive and relatively selective reduction in health care coverage and increasing inequality among individuals and groups and between urban and rural areas; More intense pressure on health care workers (especially medical doctors) to increase their productivity; and A growing gap between the demand for personalized services and standard health care provision, which calls for innovative organizational developments.
Craddock et al. 2004	The co-operative model has great potential as it fosters strong partnerships between consumers and health care providers in the design and delivery of health care services It inspires citizens to support their own health care and the health of their communities using a client-centered, holistic, and interdisciplinary approach to health care.	
Naylor et al. 2017	They are owned by their membership and therefore should be more accountable They have the potential to put a halt to the over-collection of personal data through They have data policies that reflect the wishes of their membership They can form around single issues or scale with many data subjects; and they can help their membership understand how their data is used – i.e. improve data literacy	Conﬁdentiality, privacy and data security.
Terry K. 2015	Collaborative records will be useful so that patient can access data from anywhere	Resources are needed to educate people about their data to ensure protection and security of their data.

INTERNET SEARCH		

A healthcare Startup to provide patient perspective adopts co-op model, https://medcitynews.com/2018/04/healthcare-startup-provide-patient-perspective-adopts-co-op-model	Centered on giving patients the ability to connect with the company pitches that interest them.	Fee for membership
Are digital data co-ops an alternative to the commercialisation of health? https://thisisnotasociology.blog/2017/02/27/are-digital-data-co-ops-an-alternative-to-the-commercialisation-of-health/	Individuals will be able to refuse access to their data to organizations or companies and the membership will be able to vote on who is accepted as a client.	Dangers to having central repositories for all data. Certainly, if they did become the main repository for health data they will be a very big target for attacks by hackers
Data Cooperatives - P2P Foundation	Cooperative structures could enable the creation of open data and personal data stores for mutual benefit; they could rebalance what many perceive as asymmetric relationship between data subjects (people with personal data) and data users	Legislation in many countries offers a framework about how personal data is used and shared amongst organizations, but these don’t necessarily create a mechanism that allows users to retrieve their data and use it for other purposes
Holland Health Data CooperativE, LinkedIn https://www.linkedin.com/company/holland-health-data-cooperative	The members determine what happens to their data Data is stored securely and only encrypted and released for use by third parties after your permission The cooperative does not have a profit motive. The cooperative wants to reinvest the proceeds in biomedical research that the cooperative - its members - consider important
midata.coop - Swiss Data Alliance	Governance: cooperative form, not for profit, citizen-controlled, with an ethics committee Functionality: Data entry and import, storage, visualization through web and mobile apps, sharing with friends, health professionals and researchers
MIDATA.COOPs – Personal (Health) Data Cooperatives https://www.midata.coop		Produce data in incompatible silos Secondary use of data is subject to data protection laws
Patient Ownership creating the business environment http://www.ourhdc.com/files/77395669.pdf	Patient realizes an Economic Benefit of anonymously sharing, and aggregating their health data with other members. Outcomes of various treatment options for diseases can be more quickly dispersed to patients Health Providers and Patients in all areas of the country can have access to the best scientific, valid, nonbiased and non-confounded outcomes for each disease treatment, regardless of socio-economic background
The future of your health data http://maneeshjuneja.com/blog/2014/2/12/the-future-of-your-health-data		Building trust takes time, unless you partner with an existing brand that is already trusted Privacy, security & governance. Do we have the technology in place to genuinely keep our personal data private & secure in these emerging platforms? Another issue is going to be accuracy, especially with health data that can be generated using wearable technology. What about Open Data? Some people argue that these new sources of health data should be donated into a commons, free for researchers to use for the benefit of humanity
The Personal Data Economy – A Cooperative Approach - Inspire2Live http://inspire2live.org/wp-content/uploads/10.-Ernst-Hafen-Personal-Data-Economy-a-cooperative-approach.pdf	A safe and secure platform on which people can store, manage and actively share data on their terms A not-for-profit cooperative organizational structure of the personal data platforms so that they are owned by the citizens Revenues from citizen-controlled secondary use of data are invested in projects and services that benefit members and society at large
This co-op lets patients monetize their own health data - Fast Company https://www.fastcompany.com/90207550/this-co-op-lets-patients-monetize-their-own-health-data	“When people become members, they have a voice in what we do, and they also share in our profits,”	Any patient who wants to become a Savvy member pays a buy-in fee of $34
Towards a European Ecosystem for Healthcare Data – Digital https://digitalenlightenment.org/system/files/def_healthcare_data_management_report_oct2017_v3.5.pdf	Data is controlled by citizens and patients themselves, who rely on their cooperative for support	But the more personal data is combined, the easier it is to re-identify a profile and the more difficult the anonymization process becomes
Data Commons Cooperative https://datacommons.coop/	Co-op greases the flow of data between communities in the cooperative, solidarity, new, call-it-what-you-will economy
Digital Health CRC - Home https://www.digitalhealthcrc.com/	Create a new digital workforce through at least 1000 new jobs in digital health and related industries Empowering consumers Improving understanding of health risks in individuals and communities Supporting clinical practice

## Results & Discussions

We present our results separately for our two research streams: 1) academic database and grey literature search; and 2) internet search. We start by discussing the descriptive characteristics of the records found by these two search streams. Next, the benefits and challenges of HDCs are discussed in the context of three unifying themes that have emerged from the data: 1) data ownership and control; 2) data security; and 3) data flow and infrastructure.

### Search Results

Our database and grey literature search yielded nine hundred and one records, which underwent two levels of screening, first being title and abstract screening and the second being a full text reading, to be included in the final synthesis. The final number of records that were read in detail for data abstraction was twenty-two (Figure 1). For the internet search, we initially identified eight hundred and seventy webpages across the multiple search engines. After the removal of duplicates (either internal, or external corresponding with the database and grey literature search) and a screening of the landing-page for the inclusion and exclusion criteria, thirteen webpages were included in the final analysis (Figure 2).

**PRISMA Flow diagram for search of health data co-op literature using published and grey literature databases:**
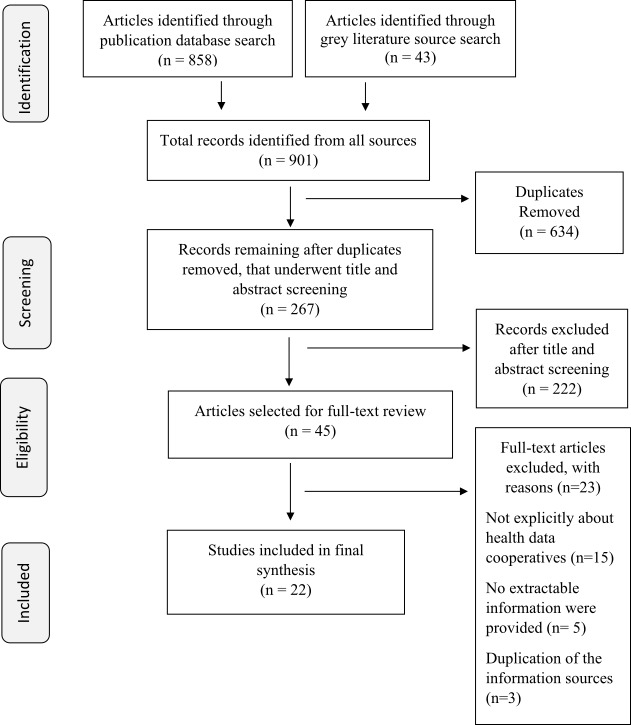


**PRISMA flow diagram for search of health data co-op literature through Internet scan:**
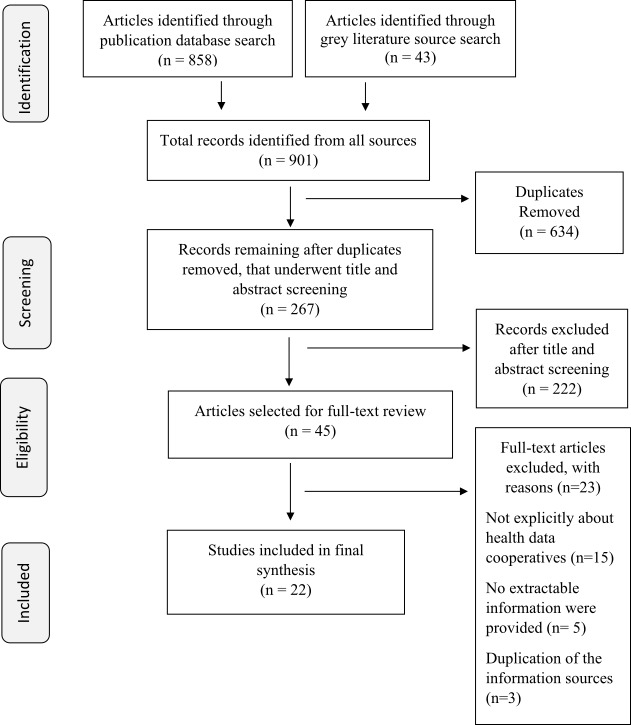


### Study Characteristics

Of the twenty-two records identified in the initial search stream, eight were grey literature records, and fourteen were academic literature published in journals. Of these twenty-two, no records were published before the year 2000, whereas records increased after the year 2014. This is expected as HDC as a topic is relatively unexplored, and technological advances leading to increased data will rightly increase the interest in study of the HDC model. Only two records were written by authors with a non-academic affiliation. Much of the target audience seems to be academia, policy, and public health workers, or simply for academia. The majority of the records originated from the United States, with Europe in second place. Only one record was identified from Canada (Table 5).

**Table 5: Descriptive characteristics of health data co-operative literature identified through published and grey literature search:** 

		All records (Grey, database, and web search)	Grey literature	Academic literature
		N = 22	(n=8)	(n=14)

Publication date	
	Earlier than 2000	0	0	0
	2000 - 2013	4	2	2
	2014 - 2016	6	2	4
	2017	5	2	3
	2018	7	2	5
Author affiliation	
	Academic	16	4	12
	Non-academic	6	4	2
Study Location	
	Canada	2	1	1
	US	11	4	6
	Switzerland	6	2	4
	Netherlands	2	0	2
	UK	2	1	1
Target Audience	
	Academia	7	3	4
	Policy	9	4	3
	Academia, policy	4	1	2
	Academia, policy, public health workers	2	0	5

Of the thirteen internet search webpages identified, nine were of a private nature (e.g. blogs, news sites, opinion pieces, corporate sites), and four were of an academic nature (authors were affiliated with an educational institution) (Table 6). The main purposes of these webpages included: 1) homepages of the sites for HDC companies; 2) academic research on the HDC model, such as conference presentations or professional opinion pieces. A significant number of the private webpages were for lay audiences, whereas all the academic records were for academic audiences. Most of the private webpages originated from the United States and Europe, with none being from Canada. All academic webpages were from Europe.

**Table 6: Descriptive characteristics of health data co-operative literature identified through Internet scan:** 

		All Records	Private	Academic
		(N=13)	(n=9)	(n=4)

Purpose of Website	
	Research	5	1	4
	Corporate	5	5	0
	Blog	2	2	0
	News	1	1	0
Target Audience	
	Professionals	2	2	0
	Academic	5	1	4
	Lay	6	6	0
Location	
	US	5	5	0
	Canada	0	0	0
	Australia	1	1	0
	Europe	7	3	4

### Major Themes and Benefits and Challenges of HDC

The HDC model seems to fill significant gaps in health data, health research, and community health. All records identify in their content that HDCs are citizen owned and the equal property of members, where the model aims to enable meaningful collaboration and facilitate a transformation of community health data [8]. The records point to integrations and merging of new types of data (i.e. lifestyle data, quantified self-movement, demographic characteristic, genetic data) that can drive improvements in health and healthcare by increasing the accuracy, accessibility, and utility of patient information [18]. Through thematic analysis, three major themes capture the data available on HDCs: 1) Data ownership and control; 2) Data security; and 3) Data flow and infrastructure. The benefits and challenged are discussed within these themes.

#### Data control and ownership

The grey literature and database search records emphasised the benefits of control that participants will have over their health data. HDCs can operate with minimal costs and without charging their participants, since the valuable nature of the data can be invested in different industries by the participants to generate profits [18,19] . This transaction can involve multiple stakeholders including primary care physicians, beneficiaries, community members, health departments, social services, and universities. Some HDC models are able to provide and gain consent electronically and build a democratic structure, which establishes a link between control and ownership [20,21]. Ideally, participants of the co-operative can have control of data access, data use, and governance. Data control, in this case, is conducive to transparency, accountability, and trust [20,22]. The web search results looked at data ownership and control from a practical perspective, since most records were from HDC companies, mainly opinions or news blogs. Here, the HDC corporations not only give patients control of their data, but also allow them to connect and pitch to interested stakeholders. Further, there seems to be a big push from private cooperatives to take control of data away from corporations and put it back into the hands of people [23,24].

Negative aspects of data control and ownership were focused on the political and bureaucratic hurdles to truly make data belong to participants. This includes: first, establishing legal precedence, where health information as a private property of patients is hard to justify based on traditional labour theory of ownership [25-28] . Second, the HDC model would need to establish connections within government agencies, which may risk the privacy of participants if robust data governance is not in place [19,23]. For example, HDCs in countries hosting public healthcare systems may need to establish health data sharing procedures that follow the countries laws and policies. The web search results took a practical and patient-centered approach to these issues. The records show there is an inherent variability in a community’s vision on how they want to use the data within the cooperative. This variability can be in who has access to the data, who is the data controller, and who outlines the data sharing agreements. This can lead to distrust, lack of respect, and insufficient patient control of the process [22,29,30].

#### Data security

Data security was equally a strength of the HDC model as much as it was a weakness. Briefly, the grey and database literature results found that HDC models are able to securely store data from multiple platforms within secure servers and cloud computing. This not only provides a financially sound solution but offers an opportunity to collect data that is comparable. For example, the creation and management of a single server to hold information can be designed such that the information is ready-to-use by interested stakeholders [31]. However, there are concerns that no technology exists thus far that can absolutely guarantee trust, transparency, and data security [32] . Although a singular space to store data provides its benefits, it is also open to security threats [33,34]. Insufficient transparency may discourage patients from entering a cooperative due to the fear of an anonymity breach.

The web results show the use of government regulatory bodies and governance structures that improve data security and transparency related to access and control of patient data, which can legitimise the HDC operation. However, considering large data repositories and their value in the market, cyber-attacks are a real threat [28,35]. This is especially true due to the ability of many cooperatives to access aspects of data through multiple modalities, including phone applications. As the access points to data repositories increase, so do security threats, and the potential costs to secure such repositories [36,37]. Indeed, to ensure data security, the HDC models must establish access and privacy standards that are communicated to HDC members and that may need to be regulated by not only good governance structures, but also independent government regulatory bodies [22].

#### Data flow and infrastructure

Records discussed data flow and infrastructure of the HDC model on multiple levels. Grey and database literature discuss the advantages of HDC in that they can create longitudinal health data from various care settings, which can be accessed via mobile applications [38]. Data integration is a highly cited strength of the HDC model in both search streams. Integration allows the use of data from multiple modalities, reducing the costs of gathering and third-party consent. Cloud computing would then allow access to the data at anytime [19,20,39]. The web results break down the process of data collection and integration. Patients can collect, store, and manage data that is lifestyle (e.g. running times, blood pressures, sleep time, number of steps taken in a day) or medical (e.g. lab results, genetic information) in nature and can input it using a simple interface on either a phone or computer. The data is then used by interested stakeholders and invested into projects that will benefit the community of patients [40-42].

The challenges to the data flow and infrastructure of the HDC include clashes with the socio-political system in the host country. For example, it is difficult to know the fate of a HDC in a publicly funded healthcare system [43]. Further, the HDC may function better with the patient population representing homogenous strata, which may include participants with similar disease profiles and risk factors, or similar demographic characteristics. If this is not the case, certain disease groups may take leading roles in decision making, domineering the voices of another legitimate cooperative stakeholder [44-48]. Finally, although data integration is an important strength of the HDC model, webpage records show that there is a risk for collecting too much data. Not only will this create waste, as some data may be unusable, but it can also make it difficult to maintain anonymity. As more personal data is combined for patients, the easier it becomes to re-identify a patient profile [49-51].

### Implications and Future Work

The work presented here has implications to inform the development of HDCs in communities that face disparities in healthcare access, health outcomes, and exposure to social cultural, and environmental influences. The key question in health research is to understand how immigrant communities fare in comparison to the health of people born in Canada. To answer this, data on long-term health outcomes, preventable conditions, and chronic disease outcomes are essential. This review outlines the political, financial, and humanistic barriers that must be overcome to establish HDCs. First, there is a need for a transparent infrastructure of an HDC that works well with the political situation in Canada, which hosts a public healthcare system. The collection and hosting of health data must overcome barriers in costs, legality, and privacy and security that must not only be effective, but also be effectively communicated to HDC members. Finally, the investment of health data must establish a democratic process where cooperative members establish informed consent, and members have a voice within the sharing of such data. Regardless of these barriers, the HDC models would encourage the development of information sharing hubs or community-based labs for immigrant communities to understand the worth and consequences of health data and serve as a source of grassroots action to understand health data. Next steps to understand HDCs further, along with the nuances of establishing an HDC, could be to conduct an in-depth systematic review, environmental scans, and content analyses of prominent HDC models. Cooperatives are ultimately a community-level initiative; therefore future work should involve community members in the exploration for HDCs in order to provide effective ready-to-use evidence.

### Strengths and limitations

This review is the first of its kind to map the literature on HDC and related topics. It has multiple strengths, the first being an extensive search strategy that includes database, grey literature, and internet search streams. The review was also strong in collating the literature into comprehensive themes that represented the data appropriately. However, this review had multiple limitations. The inclusion criteria may have been broad; however, this is something the authors felt was necessary to capture information on HDC and related topics appropriately. Further, the review was unable to assess the quality of the literature, due the variability in the type of records identified in this review. Finally, most records found hailed from Europe and the United States which present different sociopolitical and health-related environments. In this case, the results of this scoping review should be generalised to Canada with caution.

## Conclusions

The results of this extensive scoping review found HDC to be a relatively unexplored topic, with a focus in records from the United States and Europe. Canada seems to be lacking in its use, discussion, or research of the HDC model. We found that the benefits and challenges of HDC operate around three main themes related to data control, data security, and data flow and infrastructure. The study of HDC is multi-disciplinary, with themes in law, ethics, medicine, and public health, and presents a way to revolutionise the collection, storage, and use of health data that may be more sustainable than other models. The results of this study are an informative first step to the study of the HDC model, or an establishment of a HDC in immigrant populations.

### Ethics

Ethical approval is not required as this paper is research based on review of published/publicly reported literature.

## Supplementary Appendices

Supplementary Appendix 1. PRISMA reporting checklist for scoping reviews (PRISMA-ScR)

Supplementary Appendix 2. Medline search strategy
